# Pan-cancer polygenic risk score associates with cancer susceptibility following kidney transplantation

**DOI:** 10.1172/jci.insight.198098

**Published:** 2026-04-23

**Authors:** Jarmo Ritari, Kati Hyvärinen, Kirsi Jahnukainen, FinnGen Consortium, Jukka Partanen, Ilkka Helanterä, Timo Jahnukainen

**Affiliations:** 1 Finnish Red Cross Blood Service, Helsinki, Finland.; 2 Division of Hematology-Oncology and Stem Cell Transplantation, New Children’s Hospital, Helsinki University Hospital and University of Helsinki, Helsinki, Finland.; 3 The FinnGen Consortium is detailed in Supplemental Acknowledgments.; 4 Transplantation and Liver Surgery, Helsinki University Hospital and University of Helsinki, Helsinki, Finland.; 5 Department of Pediatric Nephrology and Transplantation, New Children’s Hospital, Helsinki University Hospital and University of Helsinki, Helsinki, Finland.

**Keywords:** Nephrology, Oncology, Cancer, Genetic risk factors, Transplantation

## Abstract

**BACKGROUND:**

Cancer accounts for over 20% of late posttransplant mortality, yet the contribution of genetic susceptibility to posttransplant cancer risk remains unclear. This study investigates germline genetic risk factors for posttransplant cancer in the Finnish population using data from the FinnGen cohort.

**METHODS:**

A pan-cancer polygenic risk score (PRS) was constructed using genetic variants identified in UK and US populations to assess the influence of common germline variants on time to first cancer diagnosis in 1,802 Finnish kidney transplant recipients (KTRs), of whom 317 developed posttransplant cancer. The PRS was first validated in the FinnGen non-transplantation cohort and subsequently applied to KTRs, with replication in lung and liver transplant recipients (*n* = 476). Functional relevance was explored by assessing associations between the PRS and expression levels of 2,923 plasma proteins in the UK Biobank (*n* = 53,013).

**RESULTS:**

Compared with a matched non-transplantation cohort (*n* = 68,294), KTRs exhibited earlier cancer onset. The PRS was significantly associated with time to first cancer diagnosis in the non-transplantation population (HR 1.04, 95% CI 1.038–1.056, *P* = 3.75 × 10^–25^). Among KTRs younger than 40 years, higher PRS was associated with earlier cancer onset (HR 1.08, 95% CI 1.01–1.17, *P* = 0.036), indicating a stronger genetic effect at younger ages. The PRS significantly (Bonferroni < 0.05) altered the regulation of 87 plasma proteins, several of which were known cancer-related markers.

**CONCLUSION:**

Inherited genetic predisposition, captured by pan-cancer PRS, may contribute to individual susceptibility to cancer after solid organ transplantation, particularly at younger ages.

**FUNDING:**

State research funding (Helsinki and Uusimaa Health District), the Foundation for Pediatric Research, and the Sigrid Jusèlius Foundation.

## Abstract 2

This study shows that patients with cancer diagnosed after a kidney transplant, especially at a young age, have a higher prevalence of cancer-predisposing gene variants than patients without a cancer diagnosis.

## Introduction

Solid organ transplantation (SOT) is the treatment of choice for severe kidney, liver, and heart failure, with substantially improved short- and long-term outcomes (1–3). Lifelong immunosuppression is essential to prevent allograft rejection. In pediatric and young adult patients, exposure to immunomodulatory drugs may span several decades (4, 5). Consequently, SOT recipients face increased risks of posttransplant complications, including diabetes, osteoporosis, cardiovascular disease, fertility problems, and malignancies (6–9).

Cancer is among the most serious long-term complications after SOT, accounting for over 20% of late posttransplant deaths (10–18). Large population-based studies show that transplant recipients without preexisting cancer have approximately double the risk of malignancy compared with the general population (10–15). Notably, Webster et al. reported that kidney transplant (KT) recipients are diagnosed with cancer earlier than non-transplanted patients (19). This elevated risk is largely attributed to prolonged immunosuppression (10, 20), which impairs immune surveillance and increases susceptibility to infection-associated cancers such as non-Hodgkin lymphoma, liver cancer, and Kaposi’s sarcoma. Risks of non–infection-related cancers, including kidney and thyroid cancer, are also elevated. Moreover, immunosuppressive agents like calcineurin inhibitors and azathioprine may promote carcinogenesis via non-immune mechanisms (10).

The contribution of genetic factors to posttransplant cancer risk remains unclear. Some evidence links genetic polymorphisms, especially those affecting skin type, to elevated skin cancer risk in SOT recipients. Specific variants in IL-10 and TGF-β genes have been associated with posttransplant lymphoproliferative disorder (21, 22). Stapleton et al. demonstrated (23) that a polygenic risk score (PRS) for non-melanoma skin cancer (NMSC) can predict both risk and timing of NMSC after transplantation. Another study showed (24) that PRSs can stratify SOT recipients into high- and low-risk groups for basal cell carcinoma (BCC) and squamous cell carcinoma (SCC). PRSs have also been applied to posttransplant kidney function (25) and diabetes (26). However, large-scale studies on genetic cancer risk in transplant recipients remain lacking.

**Figure 1:**
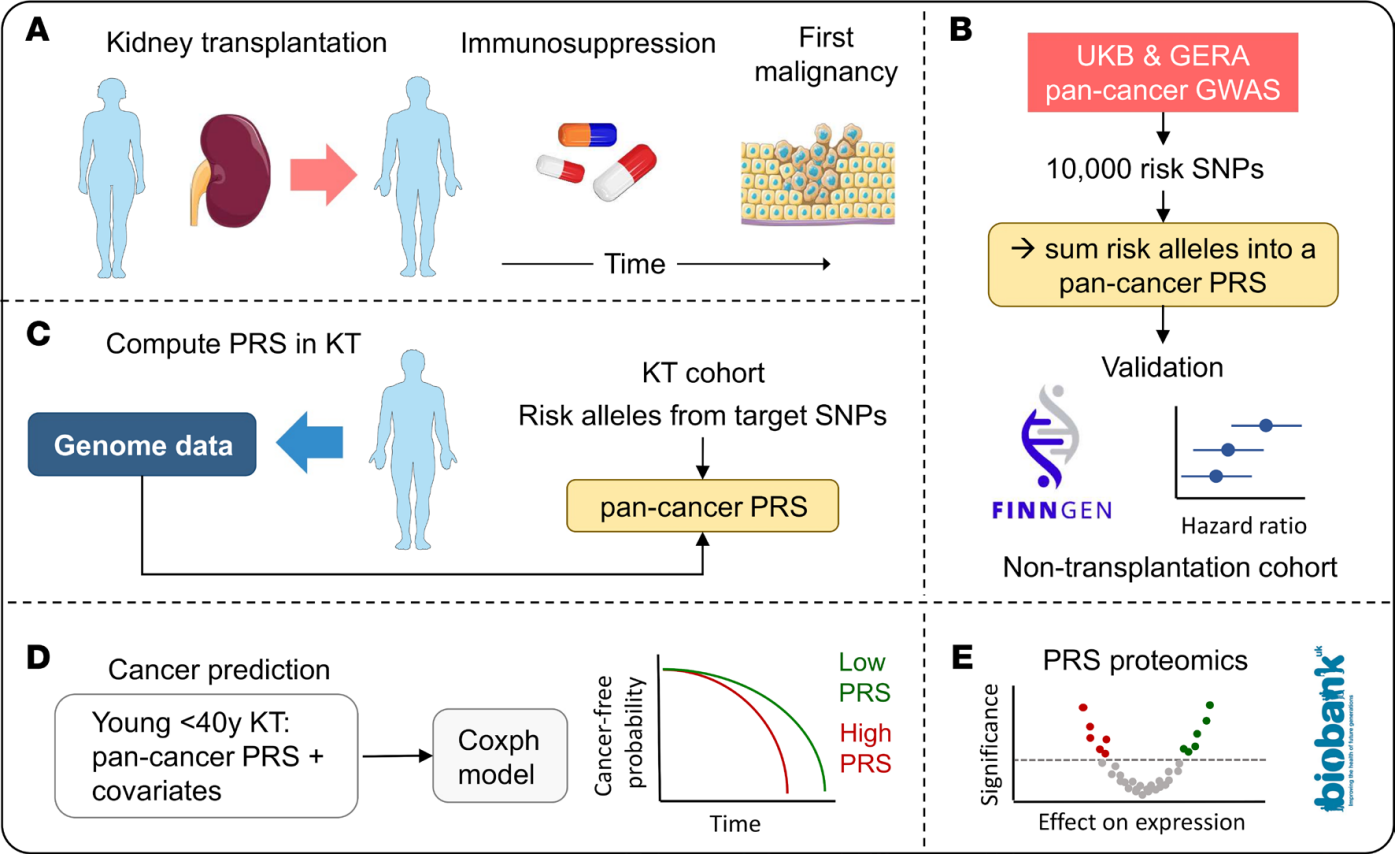
Schematic overview of the study. (**A**) Kidney transplantation (KT) patients receive long-term immunosuppression, which elevates post-KT cancer risk especially in young patients. (**B**) A pan-cancer polygenic risk score (PRS) is constructed by selecting statistically significant variants from an UK Biobank (UKB) and US Kaiser Permanente (GERA) GWAS meta-analysis (28) and extracting the risk alleles of the selected variants from FinnGen non-transplantation population (*n* = 496,641). The PRS is a sum of the number of risk variants normalized for cancer type. The pan-cancer PRS is then validated against longitudinal electronic health record data of cancer diagnoses by fitting a multivariate Cox proportional hazards (coxph) survival model for time to first cancer occurrence in FinnGen non-transplantation cohort. (**C**) The pan-cancer PRS target variants are extracted from the FinnGen KT cohort and the PRS is similarly computed for the KT patients. (**D**) The PRS in the KT cohort is combined with longitudinal cancer diagnosis data to fit coxph models. The ability of the PRS to stratify KT patients into low- and high-risk groups is evaluated. (**E**) The effect of the PRS on plasma protein expression levels is measured in the UK Biobank.

In this study, we evaluated genetic risk factors for posttransplant cancer using data from the FinnGen cohort (27), which includes genetic and health registry data from approximately 500,000 Finnish non-transplantation biobank participants and 2,000 SOT patients. To assess how common germline variants influence timing of first cancer diagnosis, we developed a pan-cancer PRS encompassing multiple cancer types (Figure 1). We validated the PRS in the non-transplantation FinnGen population and applied it to KT recipients to compare individuals with and without posttransplant malignancies. We hypothesized that KT recipients developing cancer at a young age would exhibit a higher genetic risk, as measured by the PRS, than those who remain cancer-free.

## Results

### Patient characteristics and data coverage.

The FinnGen non-transplantation dataset included 496,641 individuals, while the KT dataset comprised 1,546 patients. Liver and lung transplantation (LLT) patients numbered 476. Among the non-transplantation cohort, 115,917 individuals had a cancer diagnosis, and 317 KT patients and 99 LLT patients were diagnosed with cancer after transplantation. Bone mass index (BMI) and smoking data were available for 279,283 non-transplantation individuals and 688 KT patients.

Of the 10,626 quality-filtered genetic variants used to construct the pan-cancer PRS, 9,818 (92.3%) were present in the FinnGen genotype data. Variant counts per cancer type are listed in Supplemental Table 1; supplemental material available online with this article; https://doi.org/10.1172/jci.insight.198098DS1

Table 1 summarizes key demographic and clinical characteristics of KT recipients with and without posttransplant cancer, including age, follow-up time, and primary renal diagnosis. KT recipients with posttransplant cancer were significantly older at transplantation than those without cancer. Median follow-up time did not differ significantly. Glomerulonephritis and polycystic kidney disease (PKD) (Supplemental Table 2) were more common primary renal diagnoses among patients with cancer, while diabetic nephropathy tended to be more common in those without; however, the difference did not reach statistical significance (Table 1). Diabetes as a comorbidity also differed statistically significantly between groups. Distribution of primary diagnoses over time in the KT cohort is shown by Supplemental Figure 1. The most common cancer diagnosis class (Supplemental Table 3) among KT patients was BCC (Supplemental Figure 2). LTT patients’ demographic information is summarized by Supplemental Table 4.

### PRS validation and cancer risk in the KT population.

**Figure 2:**
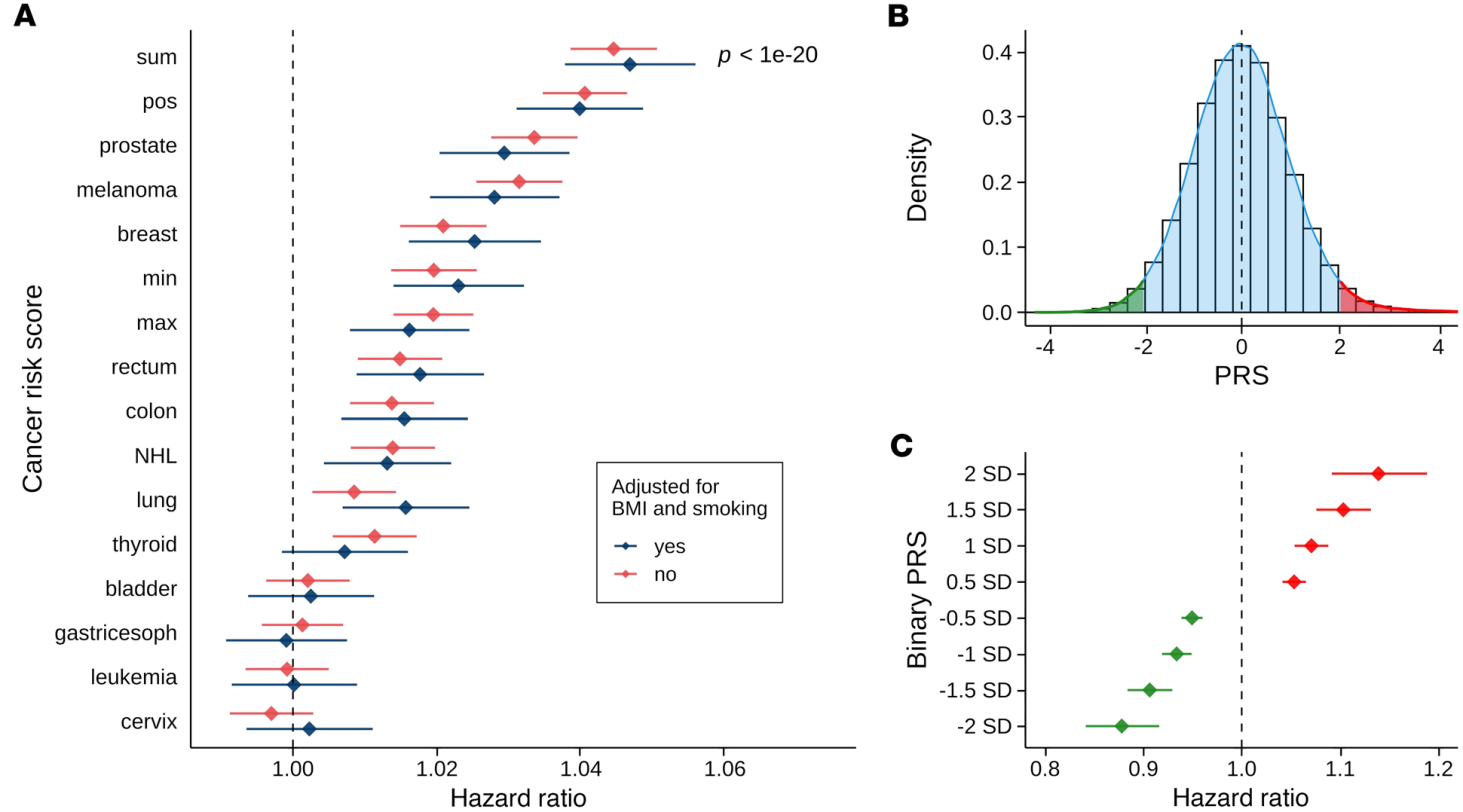
Validation of the pan-cancer polygenic risk score (PRS) in non-transplantation FinnGen cohort. (**A**) Hazard ratios for overall cancer risk of the various cancer-type-specific risk scores and the 4 pan-cancer PRS candidates (sum, max, min, and pos; see Methods for details) obtained from multivariate Cox proportional hazards (coxph) survival models. The coxph models analyze the time to the first cancer diagnosis with and without adjusting for BMI and smoking along with other model covariates such as sampling age and population stratification (see Methods). (**B**) Continuous population distribution of the best performing score (“sum”) that is used as the pan-cancer PRS of choice in the follow-up analyses. The *x*-axis unit is the standard deviation of the mean (SD). The distribution tails, which are colored according to protective (green) and risk (red) PRS directions, are most relevant for cancer risk analysis and stratification. (**C**) PRS distribution in the FinnGen non-transplantation cohort categorized into 2 classes according to SD (binary PRS) and analyzed with coxph survival models. The distribution tails behave as expected by increasing or lowering the risk according to the distanced from the PRS mean. In **A** and **C**, the error bars represent 95% CIs. Survival analyses in **A** were performed with multivariable coxph regression.

Evaluation of cancer-type-specific PRSs and 4 different pan-cancer allele scoring methods in the FinnGen non-transplantation cohort indicated that the sum of allele dosages yielded the best hazard ratio (HR) and *P* value (Figure 2A). Based on these findings, the allele dosage sum was selected as the preferred method for constructing the pan-cancer PRS. This PRS followed a normal distribution in the non-transplantation cohort (Figure 2B), allowing for stratification into low- and high-risk groups based on standard deviation (SD) thresholds. HRs for overall cancer risk, calculated using the Cox model, showed that PRS values further from the mean were associated with increasingly extreme HRs (Figure 2C), indicating a stronger association with cancer risk. Cox proportional hazards (coxph) analysis of the full dataset that included both the non-transplantation cohort and the KT cohort did not show evidence of interaction between transplantation status and the PRS (HR 1.47, 95% CI 0.6–3.5, ***P*** = 0.358) (Supplemental Table 5).

To assess the PRS’s ability to predict cancer severity in the non-transplantation group, we classified ICD-O-3 cancer behavior into benign/semimalignant versus malignant categories. A Cox model adjusting for sampling year, age at sampling, principal components 1–10, smoking status, BMI, sex, diabetes, and family history of malignancy showed a significant positive association between the pan-cancer PRS and malignant cancer (*P* < 0.001).

**Figure 3:**
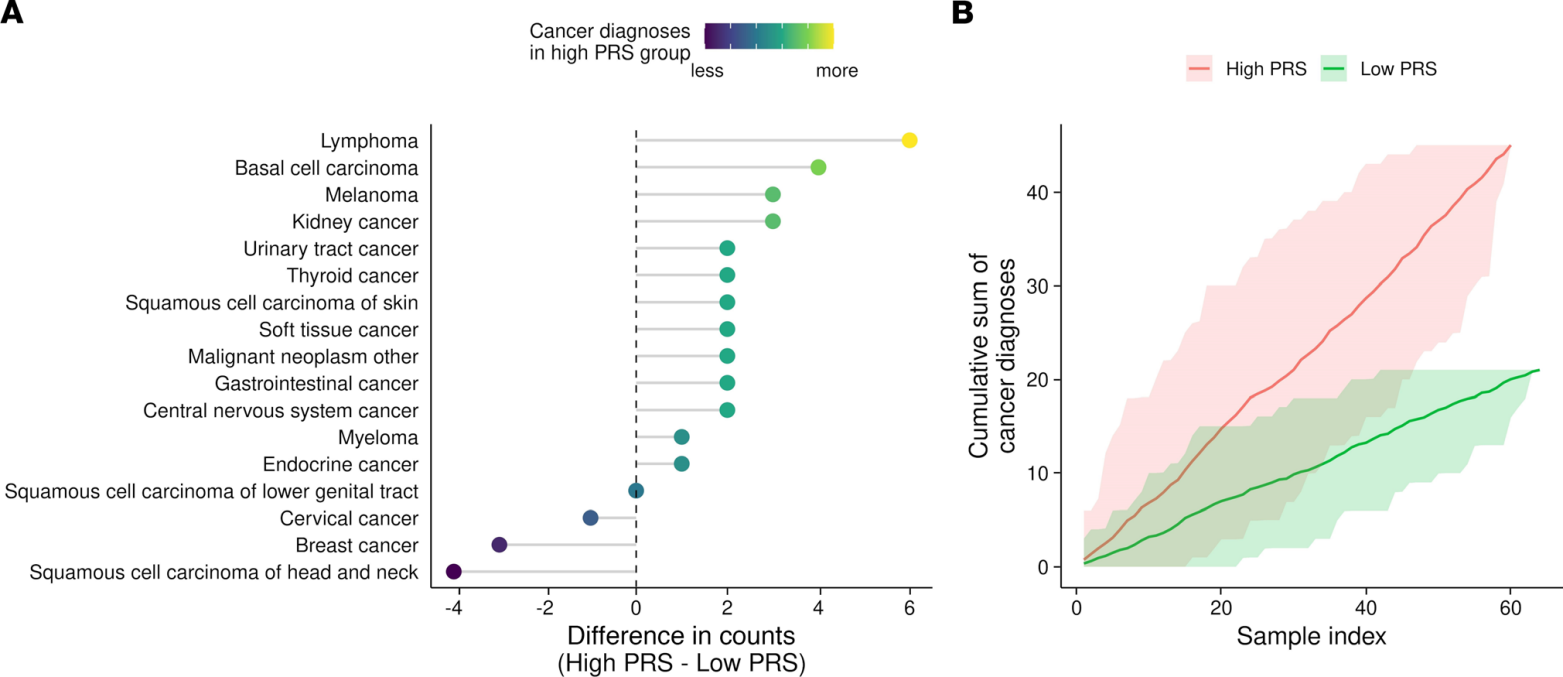
Cancer diagnoses in the high and low pan-cancer PRS groups under 40 years of age at KT. (**A**) Difference in numbers of cancer diagnoses between the high (+1 SD) and low (–1 SD) PRS groups. (**B**) Cumulative sum of unique cancer diagnoses over the follow-up period after KT. A given cancer type can be represented by multiple diagnosis entries. The lines represent averages over 100 sample order randomizations (see Methods).

In analyzing the KT and LTT cohorts, we focused on patients younger than 40 years at the time of transplantation. We hypothesized that the higher cancer prevalence in the transplant population may partly reflect prolonged immunosuppression, which could allow underlying genetic predisposition to manifest as cancer at a younger age. This age threshold is consistent with the NIH and American Cancer Society (ACS) definition of young cancer patients (<40 years; https://www.cancer.gov/types/aya). We divided the young KT patients into the high- and low-risk groups, as defined by the pan-cancer PRS ±1 SD, and analyzed their differences in terms of cancer types and number of unique cancer diagnoses. The high-risk group had more diagnoses of lymphoma and BCC compared with the low-risk group (Figure 3A), as well as a higher total number of cancer diagnoses overall (Figure 3B). However, breast cancer diagnoses were less frequent in the high-PRS group than in the low-risk group.

### Model covariates.

Analysis of the FinnGen non-transplantation cohort with coxph included age at sampling, sampling year, sex, biobank cohort, the first 10 genetic principal components, family history of malign neoplasm, and diabetes diagnosis as the basic set of analysis covariates. Race and ethnicity were not available in the FinnGen dataset and were therefore not included as variables in the analysis. BMI and smoking information were added for an additional analysis because these data were not available for all individuals (Figure 1). Family history of malignant neoplasm, representing rare high-risk germline variants, was significantly associated with increased cancer risk (*P* < 0.001) (Supplemental Figure 3), independently of the PRS.

Coxph analysis covariates for KT and LLT cohorts included sampling year, age at sampling, age at transplantation, sex, the first 10 genetic principal components, family history of malign neoplasm, and diabetes diagnosis. The KT cohort was also analyzed by including BMI and smoking as covariates, although this reduced the number of available samples (Supplemental Table 6). The most significant predictor was patient age at the time of transplantation (*P* < 0.001) (Supplemental Table 6).

### PRS and time between KT and cancer occurrence.

**Figure 4:**
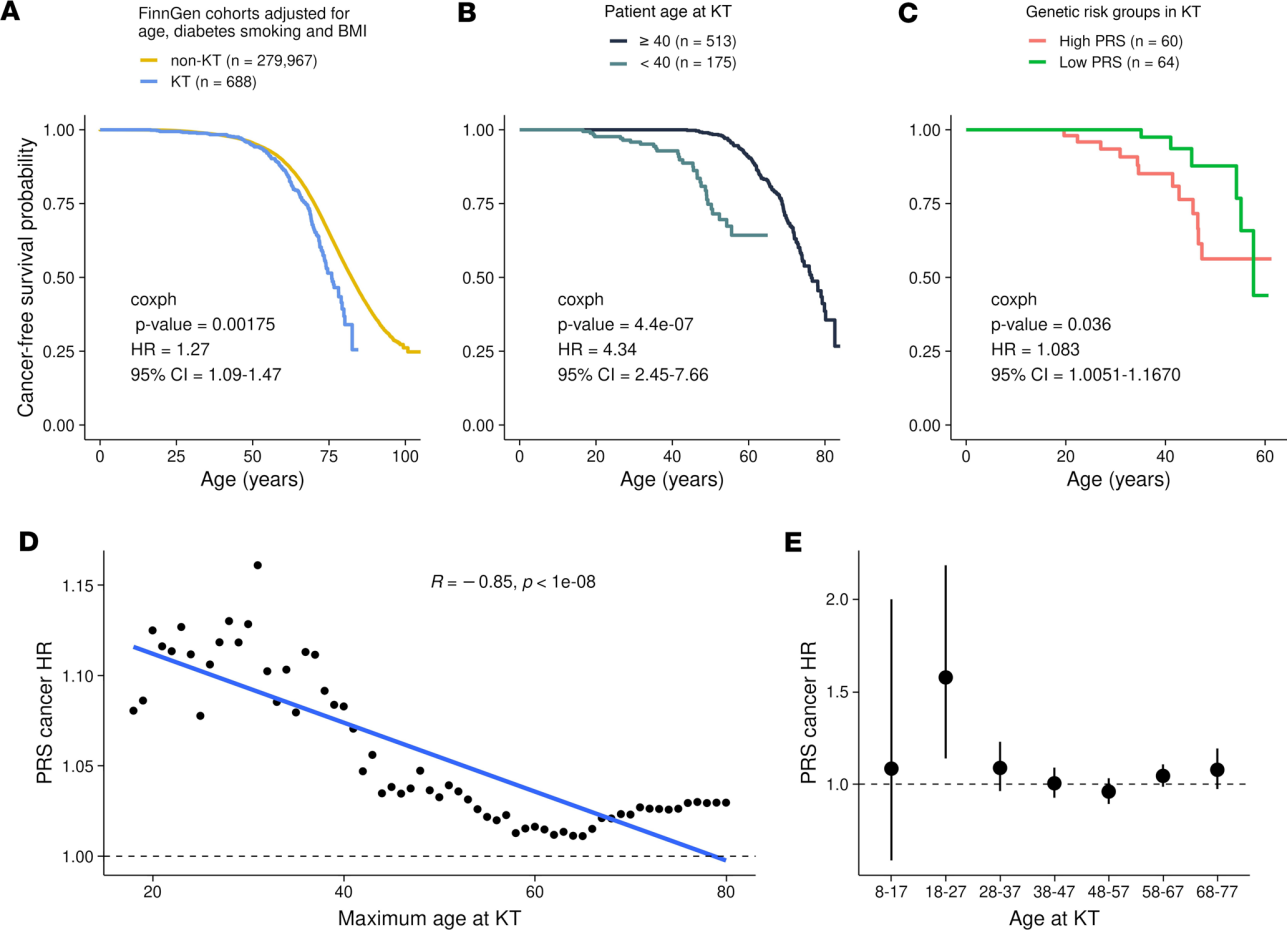
Cancer risk in KT recipients. (**A**) Kaplan-Meier survival curves for the time to the first cancer diagnosis in KT cohort versus non-transplantation cohort in FinnGen, limited to individuals sampled below 40 years of age. The *P* value was obtained from the coxph model (see Methods) adjusted for age, sex, population stratification, genetic cancer risk, BMI, and smoking. (**B**) Kaplan-Meier curves for the time to the first cancer diagnosis by binned patient age at KT. The *P* value was obtained from a coxph model as described above. (**C**) Kaplan-Meier curves for the time to the first cancer diagnosis in KT recipients under 40 years of age at the time of KT, stratified for high (+1 SD) and low (–1 SD) pan-cancer PRS. The *P* value was obtained from a coxph model for continuous PRS as described above, including transplantation age as a covariate, but excluding BMI and smoking. (**D**) Pan-cancer PRS coxph model hazard ratio for the first cancer diagnosis as a function of cumulative maximum age at KT, ranging from 18 to 80 years of age at KT. The models are adjusted as described above, including age at KT, but excluding BMI and smoking. (**E**) Pan-cancer PRS hazard ratios by KT age bins of 10 years, showing higher risk in young patients. The first bin was defined by the youngest age at KT for which the cox model could be fitted without errors. Survival analyses in **A**–**C** were performed with multivariable coxph regression. Analysis of trend in **D** was performed with univariate linear regression.

We next compared cancer occurrence between the FinnGen non-transplantation cohort and the KT cohort among individuals sampled before age 40 (the age limit of a young cancer patient according to NIH and ACS) and adjusted for age, sex, smoking, BMI, and the first 10 genetic principal components. The result showed that cancer occurred earlier in KT patients than in non-KT individuals (Figure 4A). Similarly, patient age at the time of transplantation influenced the timing of first cancer occurrence during follow-up (Figure 4B).

When stratifying KT recipients under 40 years of age at KT into high- and low-risk groups based on their pan-cancer PRS values, individuals in the high-risk group (+1 SD) experienced earlier first cancer events compared with those in the low-risk group (–1 SD) (Figure 4C). Further analysis of PRS HRs across cumulative transplantation age showed that HRs were generally higher in younger patients (Figure 4, D and E), highlighting a stronger genetic contribution to cancer risk in pediatric and young adult KT recipients. Coxph results from different age limits at transplantation for KT patients are shown in Supplemental Table 7.

### PRS in lung and liver transplantation patients.

We conducted a replication analysis of the PRS effect observed in young KT patients by applying the same PRS to LLT SOT recipients in FinnGen. In contrast to the KT cohort, the PRS was not statistically significant among LLT patients younger than 40 years at the time of transplantation (*n* = 108) when using the same covariates and including transplantation type in the coxph model. We additionally tested the interaction between the PRS and diabetes diagnosis within the same model and found that the interaction term was associated with a statistically significant increase in risk (HR 3.99, 95% CI 1.38–11.6, *P* = 0.0106) (Supplemental Figure 4).

### Adjustment for multiple testing.

After testing multiple PRS candidates in the non-transplantation cohort, assessing PRS and family history of malignancy across age groups in the KT cohort, and evaluating the PRS in the LTT cohort, and other tests, we compiled the resulting *P* values for multiple-hypothesis correction, which forms the basis of our conclusions. The Benjamini-Yekutieli–adjusted *P* values are presented in Supplemental Table 8. The corrected *P* value for the PRS in KT recipients younger than 40 years at the time of transplantation was 0.23.

### Plasma proteome quantitative traits in the UK Biobank.

**Figure 5:**
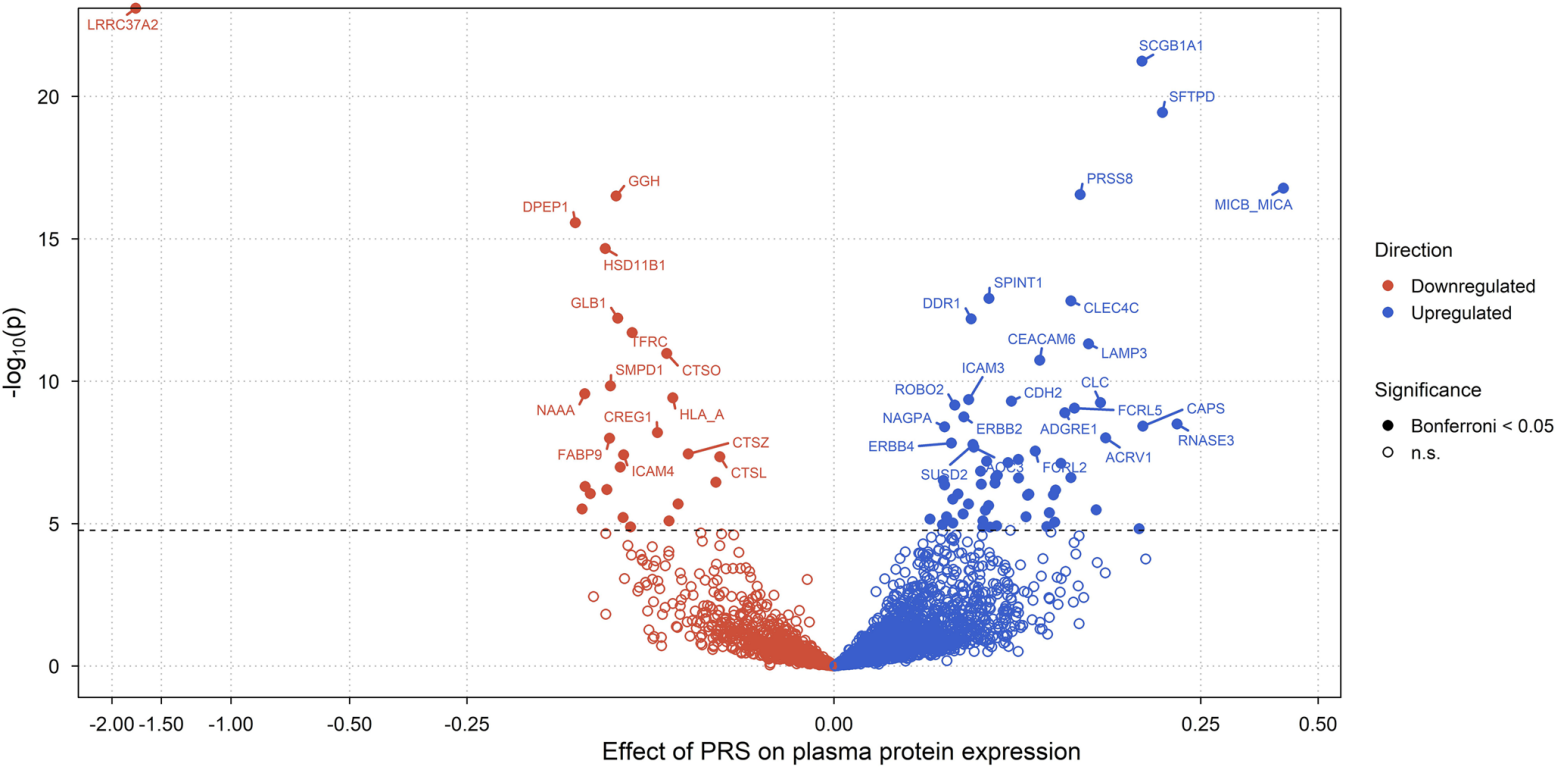
Regulation of plasma protein levels by the pan-cancer PRS in the UK Biobank. The *x*-axis displays the effect size of the PRS on plasma protein levels, and the *y*-axis shows the corresponding association *P* values. The horizontal dashed line marks the Bonferroni-corrected significance threshold (*P* < 1.71 × 10^–5^). Analysis of expression levels was performed with multivariable logistic regression.

To elucidate the functional basis of the PRS, we examined its association with plasma protein expression levels in the UK Biobank cohort (*n* = 53,013). This analysis identified 87 proteins whose levels were significantly regulated by the PRS after Bonferroni correction (adjusted *P* < 0.05; Figure 5). Of these, 61 proteins were upregulated and 26 were downregulated. We then performed functional enrichment analyses separately for the upregulated and downregulated sets (Supplemental Figure 5). Upregulated proteins were predominantly enriched for cell surface components (FDR < 0.05), whereas the downregulated proteins were enriched for lysosomal hydrolases and proteases.

## Discussion

Cancer is a major complication after SOT and contributes considerably to posttransplant mortality (10–15,20). Transplant recipients are known to develop cancer at a younger age than the general population (19, 28). To assess the role of common germline variants in cancer risk after KT, we developed a pan-cancer PRS using GWAS data from the UK Biobank and US Kaiser Permanente cohorts (29). In the FinnGen non-transplantation population, the PRS showed good transferability and supported earlier cancer onset in KT recipients. The younger age at transplantation was associated with increased cancer risk. The PRS stratified KT recipients by risk when adjusted for clinical and genetic covariates. These results indicate that common genetic polymorphisms influence post-KT cancer risk and may have future clinical utility for risk stratification.

However, interaction between transplant status and PRS was not statistically significant in our analysis, suggesting that the influence of the PRS does not appear to differ meaningfully between transplant and non-transplant individuals in our data set. This finding further highlights the need for a more focused analysis of transplant patients, particularly with respect to age at transplantation because this variable cannot be incorporated into the interaction model, as it is absent in the non-transplantation group of the full dataset.

PRSs for specific cancers, such as breast cancer, have been successfully developed in previous studies (30). However, efforts to model general cancer risk independently of cancer type are, to our knowledge, limited. Notably, Zhu and colleagues conducted such an analysis even though their PRS was limited to data from the UK Biobank, and no validation across different populations or cohorts was demonstrated (31). To date, post-KT genetic cancer prediction has primarily focused on specific cancer types. Stapleton et al. demonstrated that a PRS for NMSC could provide predictive value for posttransplant skin cancer risk (23). In s study by Seviiri et al., a PRS generated from the general population was used to stratify the risk of BCC and SCC in SOT recipients under chronic immunosuppression across both low and high ultraviolet exposure environments (24, 32). The study showed that transplant recipients in the highest PRS quintile had more than a 3-fold increased risk of BCC compared with those in the lowest quintile (24). The present results in a more heterogeneous cohort are consistent with these observations. In our cohort, 64% of post-KT cancer patients had at least one NMSC diagnosis, which may have influenced our findings. Therefore, these findings should be validated in a larger KT recipient population with a broader spectrum of posttransplant, non-skin cancers.

Pan-cancer scoring frameworks perform better in lymphomas and BCC than in breast cancer because they rely on the assumption of relatively uniform tumor biology. This assumption holds in tumor types where key driver pathways are consistent across patients, such as BCC, which is dominated by activation of the Hedgehog signaling pathway, and many lymphomas, where B cell receptor– and NF-κB–mediated signaling play central roles (33, 34). Breast cancer, by contrast, is highly heterogeneous, with intrinsic subtypes that differ markedly at genetic, transcriptomic, and microenvironmental levels. This diversity reduces the performance of tissue-agnostic scoring systems in breast tumors (35, 36).

The risk of cancer following transplantation is likely influenced by multiple factors beyond the transplantation procedure itself. Epidemiological data from the general population indicates that individuals with diabetes mellitus have an elevated risk of developing breast, colorectal, pancreatic, and liver cancers. In contrast, diabetic SOT recipients have been reported to exhibit a lower overall cancer risk (19, 37, 38). In the current study, the prevalence of diabetic nephropathy as the primary kidney disease did not significantly differ between kidney KT recipients with and without posttransplant malignancies. However, diabetes as a comorbid condition was significantly more frequent among patients without posttransplant cancer. In LLT patients, an interaction between diabetes and the PRS showed evidence of increasing posttransplantation cancer risk. This is consistent with a recent observational study in lung transplantation patients that recorded all malignancies after transplantation and reported diabetes as a risk factor (39).

Polycystic kidney disease (PKD) has been associated with an increased risk of liver, colorectal, and kidney cancers in non-transplantation populations when compared with matched controls from the general population (40). In our cohort, PKD was significantly more common among KT recipients who developed malignancies. The incidence of glomerulonephritis was also significantly higher in the cancer group, possibly due to pretransplant immunosuppressive treatments. A recent single-center study by Massicotte-Azarniouch et al. reported that pre-KT exposure to cyclophosphamide and rituximab, but not calcineurin inhibitors or mycophenolate mofetil, was associated with increased posttransplant cancer risk (41). The relationship between primary kidney disease and posttransplant cancer risk may be confounded by higher mortality rates, particularly among diabetic transplant recipients, which could limit the time available for cancer development. Furthermore, patients with cancers related to their primary disease may have been excluded from transplantation due to preexisting cancers.

In the FinnGen non-transplantation cohort, a significant association was observed between a family history of malignant neoplasms and a shorter time to cancer diagnosis. However, due to the limited number of KT patients in our study, we lacked the statistical power to properly evaluate the effect of family history on cancer risk within transplant population, although it was included as a model covariate.

The PRS–plasma proteome association analysis highlights potential mechanisms how genetic factors may affect cancer risk. The combined upregulation of cell-surface proteins and downregulation of lysosomal hydrolases/proteases suggests a shift toward sustained receptor-proximal signaling with impaired degradative control, conditions that can lower the threshold for mitogenic and prosurvival pathways, reduce receptor downregulation and autophagic quality control, and blunt antigen processing. Functionally, this asymmetric increase in signaling input with decreased lysosomal output may favor clonal persistence, accrual of additional changes, and immune evasion, thereby elevating potential cancer risk without being independently oncogenic (42, 43). Notably, several PRS-regulated proteins have established roles in modulating treatment response. For example, γ-glutamyl hydrolase, a key regulator of methotrexate polyglutamate levels, has been linked to chemotherapy resistance and prognosis (44). The analysis also points to recently proposed therapeutic targets such as SMPD1 (45) and emerging biomarkers including DPEP1 (46). Developing a multiprotein score could enhance patient stratification beyond what is achievable with genetics alone. However, causal interpretation requires careful validation and follow-up analyses, including replication, colocalization, Mendelian randomization, and rigorous control for ancestry and other confounders.

This study has several limitations. First, potential sampling bias due to voluntary biobank participation may have led to underrepresentation of certain KT recipients and overrepresentation of cancer patients among non-transplant patients. However, in assessing the validity of the PRS in the non-transplantation group with Cox regression, we included detailed cohort information about the biobank source. These adjustments should mitigate most disease risk-derived biases in the PRS validation result. Second, the relatively small number of posttransplant cancer cases within the KT cohort limits statistical power, particularly for subgroup analyses. Third, although our analysis provides evidence for the transferability of polygenic cancer risk across populations, replication in independent cohorts is lacking. Finally, the pan-cancer PRS may not capture all cancer types with equal sensitivity. For instance, post-KT cases of SCC of the head and neck and breast cancer were less frequent in the high-risk PRS group than in the low-risk group.

### Conclusion.

Our findings confirm an elevated cancer risk after KT and support a role for germline genetic variation in cancer susceptibility. The pan-cancer PRS stratifies young KT recipients by risk, but further validation is required before clinical application

## Methods

### Sex as a biological variable.

In the present study, sex was not considered as a biological variable.

### Data acquisition and study population.

The data for cancer risk association analyses were acquired from the FinnGen data freeze R12, which consisted of genotype data from the samples of 498,187 Finnish biobank participants. The genetic FinnGen data were combined with longitudinal data from Finnish health registries, including the Finnish Registry for Kidney Diseases and the Finnish Cancer Registry. All KT recipients transplanted in Finland were identified from the FinnGen database and the information about possible posttransplant cancer was achieved by linking these individuals to the Finnish Cancer Registry within FinnGen. A total of 1,801 KT recipients were identified, out of which 255 were excluded because of cancer occurrence before KT. Of the remaining 1,546 KT recipients, 317 had been diagnosed with cancer after the KT, and 1,229 recipients had no cancer diagnosis within their follow-up time frame. The primary kidney diagnoses were classified to the main categories shown in Table 1 according to diagnosis categories listed in Supplemental Table 2. Cancer diagnoses in low- and high-risk groups in KT were classified into categories according to Supplemental Table 1.

To identify SOTs other than KT in FinnGen, we searched longitudinal data using operation codes JJC00 and JJC01 corresponding to allogenic transplantation of liver and allogenic partial transplantation of liver. Furthermore, we searched endpoint data for terms “lung transplant” and “lung transplantation.” All samples overlapping with KT or showing cancer diagnosis prior to transplantation were excluded.

### Genotyping.

FinnGen samples were genotyped with Illumina and Affymetrix (Thermo Fisher Scientific) microarrays. Genotype calls were made with Illumina Bead Studio GenCall (https://illumina-beadstudio.software.informer.com/) or zCall (https://github.com/jigold/zCall) for Illumina array data and with AxiomGT1 algorithm (http://tools.thermofisher.com/content/sfs/brochures/dmet_plus_algorithm_whitepaperv1.pdf) for Affymetrix array data. Genotype data produced with older array platforms and reference genome builds were lifted over to GRCh38/hg38. Genotype quality control criteria included removing samples with a mismatch between genetically inferred sex and the reported sex in registry data, greater than 5% genotype missingness, and heterozygosity exceeding 4 SD. Furthermore, variants with greater than 2% missingness, significant deviation from Hardy-Weinberg equilibrium (*P* < 1 × 10^–6^), and minor allele count of less than 3 were removed. Genotype imputation was performed with the population-specific SISu v.3 imputation panel comprising 3,775 Finns with 25×–30× coverage whole-genome sequencing data using Beagle 4.1 (v.08Jun17.d8b, https://faculty.washington.edu/browning/beagle/b4_1.html). Quality control of imputed variants involved a conformity test against the imputation panel and exclusion of variants with imputation INFO scores of less than 0.6 or MAF values of less than 0.0001. Details of the FinnGen project and data analysis pipeline are previously described in the FinnGen flagship paper (27).

### Genetic analyses.

The pan-cancer PRS was constructed from common polymorphisms identified by a meta-analysis of the UK Biobank and US Kaiser Permanente cohorts (https://github.com/Wittelab/pancancer_pleiotropy) (29). The summary statistics result data were filtered for variants that had a fixed effects *P* value of less than 5 × 10^–8^ and low variance between cohorts as measured by an I^2^ heterogeneity index value of less than 5 (47). Next, for each cancer type assessed by the meta-analysis (bladder, breast, cervix, colon, gastroesophageal, kidney, leukemia, lung, melanoma, non-Hodgkin’s lymphoma, prostate, rectum, thyroid), variants fulfilling the above inclusion criteria were identified and extracted from FinnGen genotype data, and converted to allele dosage format (i.e., 0, 1, or 2 alleles) using plink2 (48) (v2.00a6LM, www.cog-genomics.org/plink/2.0/) “recode A” command. The dosages were then reoriented to risk allele dosages. The risk allele dosages were averaged within each cancer type, scaled to mean zero and unit variance, and combined into a candidate PRS. Candidate PRSs were constructed in 4 different ways: (a) “sum” computes a sum over the different cancer-type-specific risk allele mean scores; (b) “max” selects the highest from the different cancer type-specific scores; (c) “min” selects the lowest from the different cancer type-specific scores; and (d) “pos” selects the cancer-type-specific scores reaching over the population mean and computes a sum of those. The scores were computed for all FinnGen individuals including the KT recipients, and the impact of the scores on time to the first cancer diagnosis were first analyzed in the non-transplantation FinnGen cohort to validate and compare the scores. Finally, the best performing score was evaluated within the FinnGen KT cohort for its ability to predict the time to the first cancer diagnosis after KT.

### Statistics.

Statistical analyses and data management were performed with R software v4.3.2 (49) (https://www.R-project.org) using RStudio v2023.03.1 (50). Coxph models for time-to-cancer analyses were performed with the R package survival v3.2-7 (51, 52) function coxph, and Kaplan-Maier plots were drawn using function survfit. Plotting and data manipulation was performed with the R package tidyverse v1.3.0 (53). Multiple testing for hypotheses addressing the role the PRS and genetics in cancer risk was performed using the Benjamini-Yekutieli procedure (Supplemental Table 8). Analysis scripts are available in GitHub (https://github.com/orgs/FRCBS/post-KT_cancer).

### Coxph model covariates.

Analysis of the FinnGen non-transplantation cohort for PRS validation with coxph included age at sampling, sampling year, sex, biobank cohort, the first 10 genetic principal components, family history of malign neoplasm, and diabetes diagnosis as analysis covariates. BMI and smoking information were added for an additional analysis because these data were not available for all individuals.

Coxph analyses comparing FinnGen KT and non-TX cohorts included diabetes diagnosis, smoking, BMI, family history of malign neoplasm, sex, age at sampling, sampling year, and the first 10 genetic principal components as model covariates.

Coxph analyses within the KT and LLT cohorts included age at sampling, sampling year, age at transplantation, sex, the first 10 genetic principal components, family history of malign neoplasm, and diabetes diagnosis as model covariates.

### Analysis of cancer diagnoses.

We compared the cumulative sums of unique cancer diagnoses recorded for each patient between the high and low PRS groups under 40 years of age at KT. Prior to the analysis we removed broad, non-informative categories present in every patient with any type of cancer diagnosis, such as “cancer” or “neoplasm,” and focused on specific diagnoses only. Since the order of patients for the calculation of the cumulative sum is arbitrary, we randomized the order of patients 100 times and computed an average of the cumulative curves over these randomizations; 95% CIs were calculated based on variation within these randomizations.

### Plasma proteomics PRS-pQTL analysis in the UK Biobank.

The pan-cancer PRS was computed in the UK Biobank using the same approach applied in FinnGen, as described above. We then merged the PRS values with plasma proteomics measurements and analysis covariates for 53,013 individuals with available proteomics data. Age at recruitment (data-field 21022), sex (data-field 22001), and the first 10 genetic principal components (data-field 22009) were included as covariates in regression models assessing the association between PRS and protein expression level. The models were fitted using the bayesglm function from the R package arm (v1.14-4). Data processing and management were performed using dplyr (v1.1.4) and data.table (v1.18.2.1). This research has been conducted using the UK Biobank Resource under Application Number 74245.

We accepted proteins below a Bonferroni-adjusted *P* value of 0.05 as statistically significant and analyzed functional enrichment of significantly upregulated and downregulated proteins separately using the STRING database (https://string-db.org/) (54) with default settings (accessed on December 22, 2025).

### Study approval.

Study subjects in FinnGen provided informed consent for biobank research, based on the Finnish Biobank Act. Alternatively, separate research cohorts, collected prior the Finnish Biobank Act came into effect (in September, 2013) and start of FinnGen (August, 2017), were collected based on study-specific consents and later transferred to the Finnish biobanks after approval by Fimea (Finnish Medicines Agency), the National Supervisory Authority for Welfare and Health. Recruitment protocols followed the biobank protocols approved by Fimea. The Coordinating Ethics Committee of the Hospital District of Helsinki and Uusimaa (HUS) statement number for the FinnGen study is Nr HUS/990/2017. The present study was approved by the FinnGen administration team (F_2023_043). Further information on FinnGen ethics approvals is available in the supplemental material.

The UK Biobank (55) is a prospective cohort study comprising genotypic and phenotypic data from over 500,000 voluntary participants aged 37–73 years at recruitment. All participants provided informed consent. For the PRS-pQTL analyses, we used plasma proteomics data generated with the Olink Explore 3072 platform as part of the UK Biobank Pharma Proteomics Project Consortium (UKB-PPP).

### Data availability.

FinnGen summary statistics data from more than 5 individuals from release 12 (R12) are publicly available at https://r12.finngen.fi/ Individual-level data cannot be made public under data protection regulations of GDPR. UK Biobank data are available for use by eligible researchers from academic, charity, government, and commercial organizations from around the world, for health-related research that is in the public interest. Analysis scripts are available at GitHub: https://github.com/FRCBS/KT_cancer All data presented in the paper, except individual-level data protected by GDPR regulations, are included in the Supporting Data Values file and in Supplemental Tables.

## Author contributions

JR, KH, JP, and TJ conceived and designed the study, JR did data analysis and statistical analysis, and JR and TJ drafted the manuscript. KH, KJ, JP, and IH participated in interpretation of the results and critically revised the manuscript, and all authors accepted the final version of the manuscript.

## Conflict of interest

The authors have declared that no conflict of interest exists.

## Funding support

- State research funding (Helsinki and Uusimaa Health District).
- Foundation for Pediatric Research (to TJ).
- Sigrid Jusèlius Foundation (to JR and KH).
- Business Finland grants HUS 4685/31/2016 and UH 4386/31/2016 (to FinnGen).
- Industry partner grants from AbbVie Inc., AstraZeneca UK Ltd, Biogen Inc., Bristol Myers Squibb (and Celgene Corporation and Celgene International II Sàrl), Genentech Inc., Merck Sharp & Dohme LCC, Pfizer Inc., GlaxoSmithKline Intellectual Property Development Ltd., Sanofi US Services Inc., Maze Therapeutics Inc., Janssen Biotech Inc, Novartis AG, and Boehringer Ingelheim International GmbH (to FinnGen).

## Supplementary Material

Supplemental data

ICMJE disclosure forms

Supporting data values

## Figures and Tables

**Table 1:**
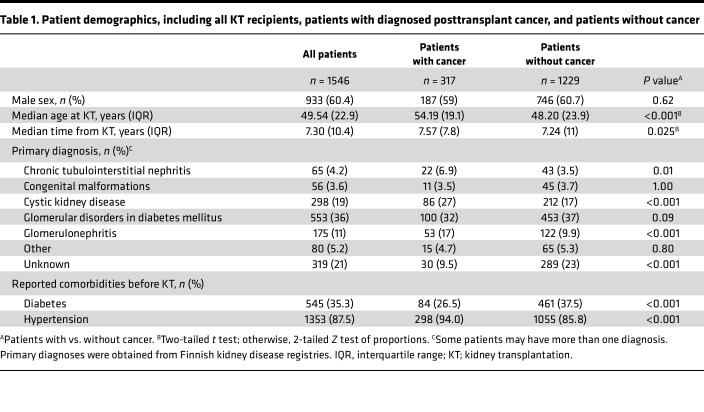
Patient demographics, including all KT recipients, patients with diagnosed posttransplant cancer, and patients without cancer

## Version 1 04/23/2026

In-Press Preview

## Version 2 06/08/2026

Electronic publication
